# Data-driven comorbidity analysis of 100 common disorders reveals patient subgroups with differing mortality risks and laboratory correlates

**DOI:** 10.1038/s41598-022-23090-3

**Published:** 2022-11-02

**Authors:** Miika Koskinen, Jani K. Salmi, Anu Loukola, Mika J. Mäkelä, Juha Sinisalo, Olli Carpén, Risto Renkonen

**Affiliations:** 1 Faculty of Medicine, University of Helsinki, Helsinki, Finland; 2 Helsinki Biobank, Helsinki University Hospital, Helsinki, Finland; 3 Analytics and AI Development Services, Helsinki University Hospital, Helsinki, Finland; 4 Division of Allergology, Skin and Allergy Hospital, Helsinki University Hospital and Helsinki University, Helsinki, Finland; 5 Heart and Lung Center, Helsinki University Hospital, and Helsinki University, Helsinki, Finland; 6 HUS Diagnostics, Helsinki University Hospital, Helsinki, Finland

**Keywords:** Outcomes research, Diseases, Diagnostic markers, Comorbidities

## Abstract

The populational heterogeneity of a disease, in part due to comorbidity, poses several complexities. Individual comorbidity profiles, on the other hand, contain useful information to refine phenotyping, prognostication, and risk assessment, and they provide clues to underlying biology. Nevertheless, the spectrum and the implications of the diagnosis profiles remain largely uncharted. Here we mapped comorbidity patterns in 100 common diseases using 4-year retrospective data from 526,779 patients and developed an online tool to visualize the results. Our analysis exposed disease-specific patient subgroups with distinctive diagnosis patterns, survival functions, and laboratory correlates. Computational modeling and real-world data shed light on the structure, variation, and relevance of populational comorbidity patterns, paving the way for improved diagnostics, risk assessment, and individualization of care. Variation in outcomes and biological correlates of a disease emphasizes the importance of evaluating the generalizability of current treatment strategies, as well as considering the limitations that selective inclusion criteria pose on clinical trials.

## Introduction

Appreciation of disease heterogeneity in a patient population is a prerequisite of and the grand goal of achieving personalized care. Heterogeneity, which is partly attributable to comorbidity, complicates both clinical practice and determination of etiological factors of a disease. Concomitant diseases represent statistical associations^1–7^, shared genetic risks, and biochemical pathways^8–15^. Such concurrent effects and systemic interactions bring variation and complexity in symptoms and outcome^16^. Therefore, longitudinal real-world data is valuable in refining phenotypes^17^ crucial for personalizing care and discovering etiology.

Comorbidity complicates interventions, predisposes to suboptimal therapies, and requires more services from healthcare systems^7,18–21^. Current therapeutic practices are often based on clinical trials that may exclude patients with comorbidities^22^ and thus lack real-world complexity. Instead of clinical presentation, much of comorbidity research focuses on relations between diagnostic codes^1,2,4,5,8,23^. This approach has revealed disease progression sequences and dependency networks between diagnoses, as well as associations between comorbidities and variables such as age, sex^4,5^, and risk of death^24^. Focusing on individuals, the burden of comorbidity has been assessed with univariate scores like Charlson^25^ or Elixhauser^26^ Comorbidity Indices. The recent focus on multivariate analysis and machine learning methodology, including clustering techniques^16,27^, has been a crucial step forward. Still, population diversity and implications of various diagnostic profiles using large-scale clinical data from everyday practice remain largely uncharted.

Here we investigated four-year follow-up data of 526,779 individuals representing the 100 most common diagnoses among 1.28 million patients in HUS Helsinki University Hospital (HUS), Finland. We wanted to examine, whether individual comorbidity profiles form population structure revealing patient subgroups, given an index disease; and whether subgroups differ in mortality risk and associations with clinical laboratory data. To illustrate the approach, we highlight two diagnoses, asthma (J45) and atrial fibrillation (I48). Comprehensive results can be searched online at https://hus100.med.helsinki.fi.

## Results

### One hundred most common diagnoses

**Figure 1:**
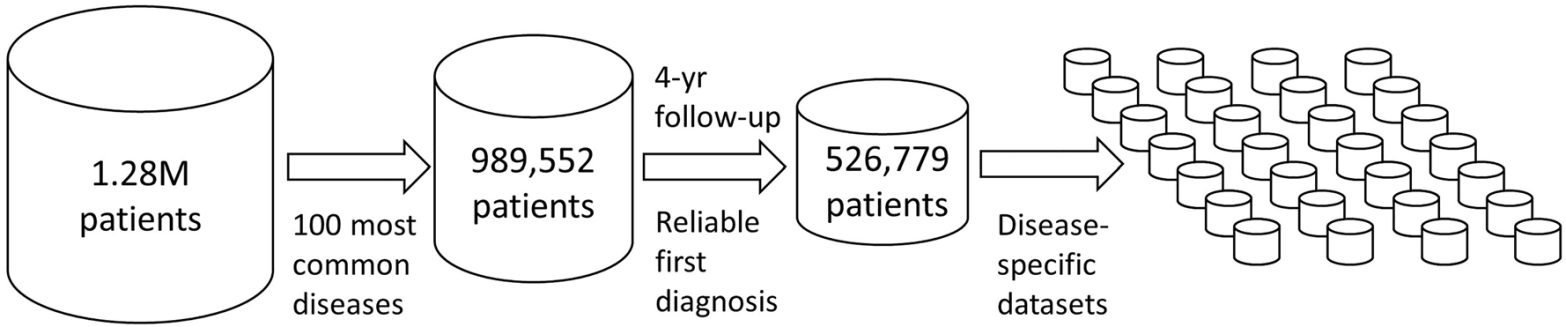
Data preprocessing scheme from original records to 100 index disease datasets.

Initially, for the 100 most frequent diagnoses found in the patient registry, we created corresponding datasets for each index disease (Fig. 1). In frequency, primary hypertension ranked first. Table 1 lists the top 30, and the full list of 100 diagnoses is provided in Supplementary Table 1.

**Table 1:** The most common ICD-10 diagnoses, with sex- and age distributions.

Rank	Code	Description	Patients	Women				Men		
				%	Age, percentiles					
					25th	50th	75th	25th	50th	75th
1	I10	Essential (primary) hypertension	87,273	53	60	69	79	56	65	74
2	J06	Acute upper respiratory infections at multiple and unspecified sites	70,515	52	2	8	35	1	4	18
3	M54	Dorsalgia [back pain]	66,696	59	34	48	62	35	48	61
4	J18	Pneumonia, organism unspecified	61,383	47	37	63	78	41	64	76
5	J45	Asthma	56,301	55	16	44	62	7	15	53
6	H25	Senile cataract	55,387	62	68	75	80	66	73	79
7	M79	Other soft tissue disorders, not elsewhere classified	54,662	60	34	50	63	30	49	65
8	F32	Depressive episode	54,168	63	16	19	37	17	25	45
9	I48	Atrial fibrillation and flutter	54,048	45	67	75	82	59	68	76
10	M17	Gonarthrosis [arthrosis of knee]	44,729	63	59	67	75	56	65	72
11	H66	Suppurative and unspecified otitis media	43,433	47	2	4	16	1	3	7
12	H90	Conductive and sensorineural hearing loss	43,345	54	34	59	73	19	58	72
13	G47	Sleep disorders	42,894	34	49	58	66	46	55	64
14	I25	Chronic ischemic heart disease	42,746	36	66	75	82	62	70	77
15	A09	Diarrhea and gastroenteritis of presumed infectious origin	40,501	54	6	32	65	3	23	55
16	F41	Other anxiety disorders	36,647	66	16	21	35	17	25	40
17	E11	Non-insulin-dependent diabetes mellitus	36,537	42	59	68	76	58	66	72
18	N39	Other disorders of urinary system	36,471	81	43	61	74	58	70	77
19	M25	Other joint disorders, not elsewhere classified	35,719	60	27	42	56	26	41	54
20	K57	Diverticular disease of intestine	33,404	59	58	67	76	53	64	73
21	M51	Other intervertebral disc disorders	33,299	54	38	48	59	38	48	57
22	I50	Heart failure	30,504	49	72	80	86	64	73	80
23	L20	Atopic dermatitis	30,063	54	5	21	39	2	8	30
24	N10	Acute tubulo-interstitial nephritis	30,058	64	19	54	76	48	66	77
25	K80	Cholelithiasis	29,707	65	42	57	70	49	63	74
26	I63	Cerebral infarction	29,281	47	60	72	81	55	66	75
27	M75	Shoulder lesions	28,915	53	47	54	61	47	55	62
28	K40	Inguinal hernia	28,275	14	35	63	76	46	62	72
29	F33	Recurrent depressive disorder	28,011	68	28	39	51	29	41	52
30	F10	Mental and behavioral disorders due to use of alcohol	26,849	33	25	42	55	35	47	57

### Comorbidity analysis

**Figure 2:**
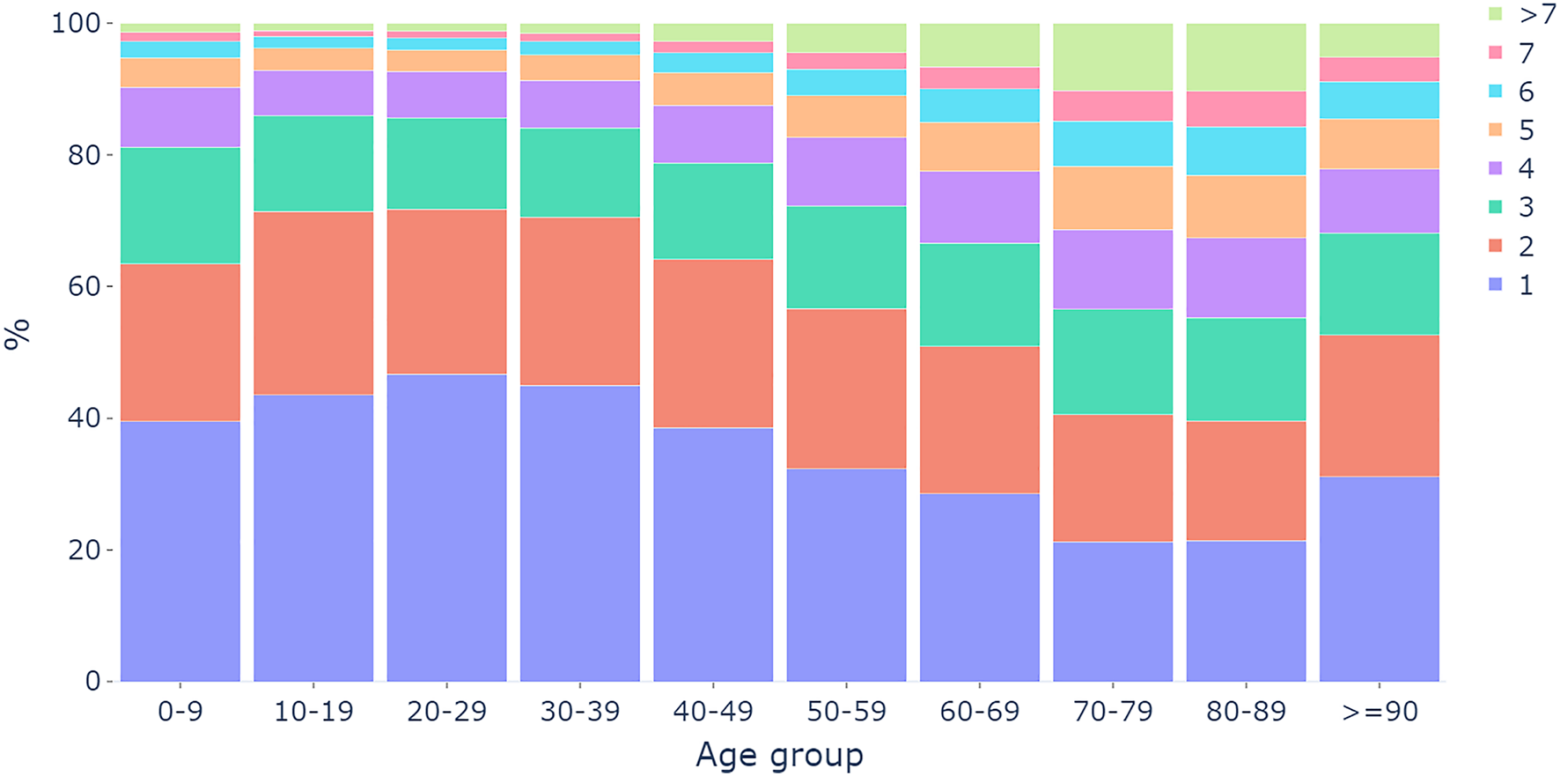
Number of differing diagnoses during 4-yr follow-up in age groups, with only diagnoses among the 100 most common counted.

Patients with multiple diagnoses during the 4-year follow-up appeared frequently. In the disease-specific datasets, 65% of patients had more than one, and 41% more than two distinct diagnoses. The number of comorbidities was largest in patients aged 70–89 (Fig. 2). Of 100 index diseases, in 99, the median diagnoses per patient numbered two or more, maximally five in disorders of lipoprotein metabolism and other lipidaemias (E78), heart failure (I50), and angina pectoris (I20). Only in the group of acute appendicitis (K35) was the median number one.

For each 100 index diseases, we mapped the variety of patients’ multivariate diagnostic profiles by a robust data-driven analysis scheme, with 2–31 patient subgroups per disease. A median 20% (0–39%) of patients were without clear cluster assignments and excluded as outliers. Reliability and associations with laboratory values and survival we computed for each cluster.

### Comorbidity in asthma

**Figure 3:**
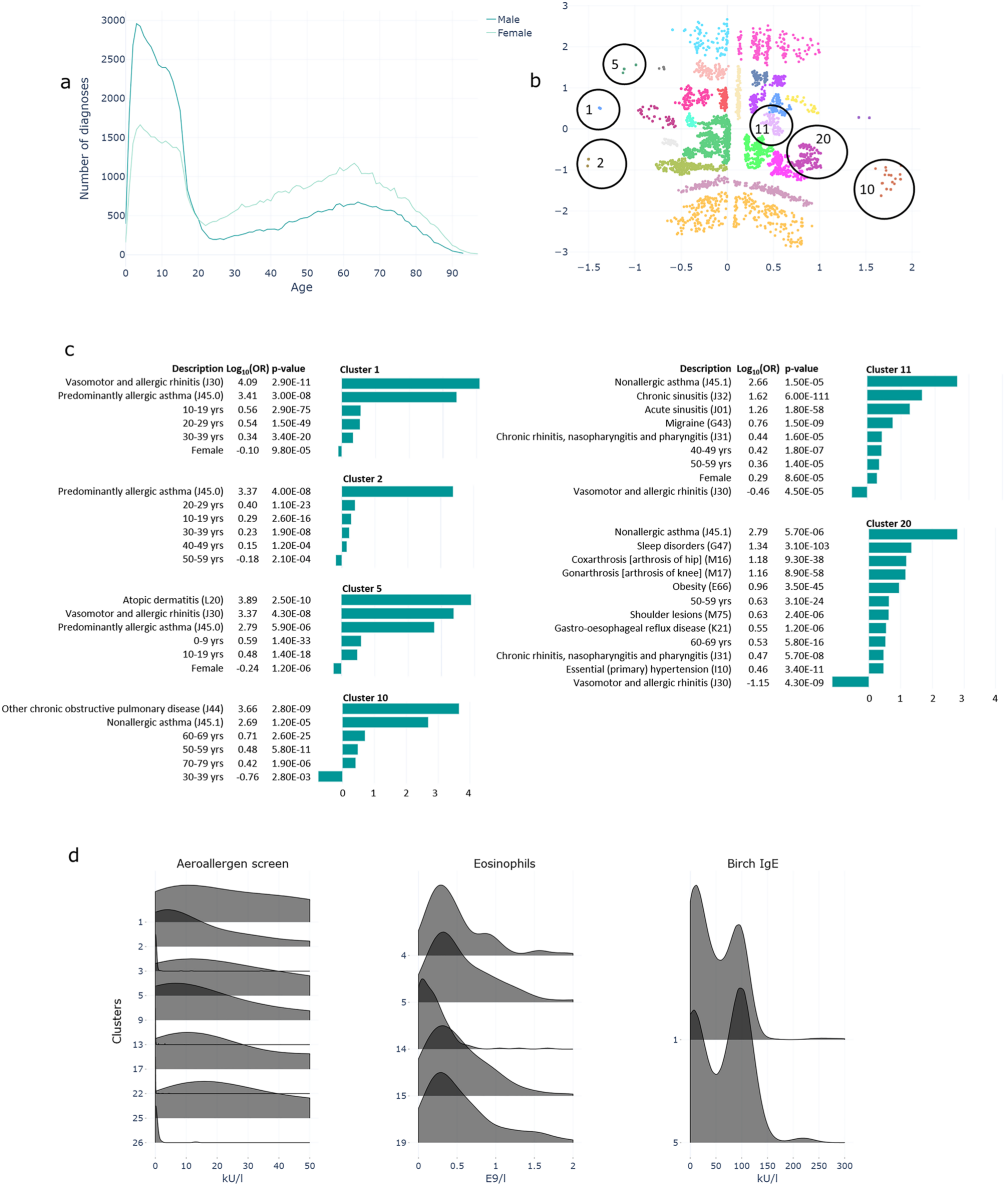
Asthma. (**a**) Age and sex-distribution of diagnoses among patients in secondary and tertiary care. (**b**) Heterogeneity of asthma patients in 27 clusters represented in two-dimensional latent space of VAE model. Clusters mentioned in the text are circled. (**c**) Cluster-specific characteristics presented by statistically significant logarithmic odds ratios for demographics and diagnoses. (**d**) Distributions of laboratory results that differ statistically significantly (FDR 0.1%) between a specific cluster and the rest of the patients (only selected tests shown).

We chose asthma (J45) as one disease example. Age-specific number of diagnoses (Fig. 3a) reflects treatment for asthma in the Finnish health care system. Treatment for children is organized mainly in specialized care. At the age of 16 to 18, patients are typically remitted to primary care services, with only the more severe cases treated at pulmonary clinics, explaining the sharp decrease at adolescence in the number of patients within secondary and tertiary care. Asthma is common across all ages but demonstrates a clear sex-dependent pattern: among patients with a new asthma diagnosis, males dominate in early childhood, but after age 15, females dominate.

Figure 3b demonstrates the heterogeneity of the asthma phenotypes in the population and in the 27 subgroups. The most common comorbidities included disorders of nasal function (rhinosinusitis, allergic rhinitis) and several atopic diseases. In clinical work, diagnosis is typically either an allergy-driven (J45.0), non-atopic (J45.1), or undefined asthma (J45). These diagnoses occurred in distinct clusters (Fig. 3). Some clusters followed the traditional allergic vs. non-allergic pattern, whereas others represented mixed asthma phenotypes. For example, Cluster 1 comprises young patients less than age 40 with rhinitis as their main co-morbidity, Cluster 2 comprises patients with allergic asthma but lower sensitization levels, and Cluster 5 patients with several atopic comorbidities including dermatitis and rhinitis with high eosinophils. Of the non-allergic asthma patients, Cluster 10 comprises patients over age 50 with a mixed phenotype of asthma and chronic obstructive pulmonary disease (COPD), Cluster 11, females over 40 with chronic rhinosinusitis, and Cluster 20, obese 50- to 70-year-old patients with sleep apnea, high blood pressure, and osteoarthritis. We detected unexpected differences between the clusters, for example, in mean corpuscular volume (MCV) of erythrocytes and in renal function measurements, these, regarding survival, likely associated with severe infections (see the online tool https://hus100.med.helsinki.fi).

### Comorbidity in atrial fibrillation

**Figure 4:**
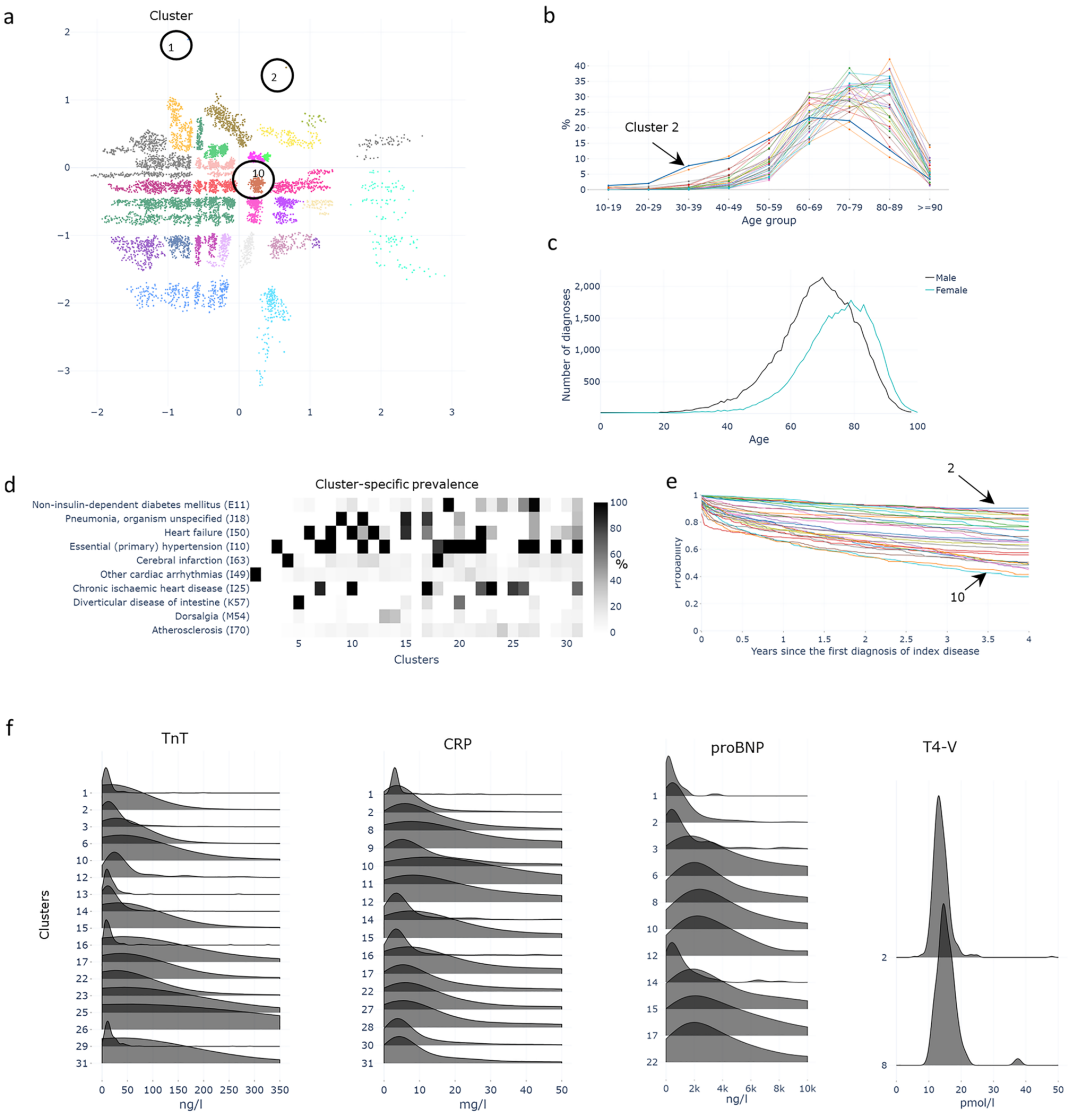
Atrial fibrillation. (**a**) Disease-specific comorbidity clusters represented in two-dimensional latent space of VAE model. (**b**) Age distribution of the clusters, and (**c**) age- and sex distributions of I48 diagnosis among original 1 M patients (**d**) The prevalence of the 10 most frequent diagnoses in comorbidity clusters shown on a heat map. Some diagnoses, e.g. other cardiac arrythmias, diabetes mellitus, and diverticular disease of the intestine, demonstrate cluster specificity, whereas pneumonia, heart failure, hypertension, and ischemic heart disease are more widely distributed across clusters. (**e**) Kaplan–Meier plot showing variation in cluster-specific survival rates. (**f**) Distribution of laboratory measurements of selected tests. Only clusters with statistically significant (FDR 0.1%) results shown.

Our second disease example is atrial fibrillation (I48), the registry’s ninth most common diagnosis. Clustering analysis resulted in 31 comorbidity subgroups (Fig. 4a)—the largest number of subgroups among all of the 100 diseases—often with distinctive characteristics such as hypertension, the males being of younger ages, or stroke. In the cohort, atrial fibrillation is notable in older age, (Fig. 4b) however, in cluster 2 the shape of the age distribution is distinctive raising very early for atrial fibrillation patients, at 30 years, and peaking also at earlier age. The overall peaking of age distribution is ten years later in women than in men (Fig. 4c). The most common concomitant diagnoses include other cardiac arrythmias (I49), heart failure (I50), hypertension (I10), sleep disorders (G47), and mental and behavioural disorders due to use of alcohol (F10), *i.e*. known causes or comorbidities of atrial fibrillation. Age distribution of concomitant diagnoses (according to age groups) showed age-dependency of comorbidities: in 20- to 39-year-olds, other cardiac arrhythmias were frequent, while in 40- to 89-year-olds, hypertension and heart failure were the most common comorbidities. Patients in Cluster 1 (Fig. 4d), on the other hand, had other arrhythmias (I49) and were of a relatively young age (30–49 years), and these had the highest survival rate (90% over 4 years). Patients in Cluster 10, for example, were characteristically diagnosed with heart failure and ischaemic heart disease and were associated with the shortest life expectancy (40% survived beyond 4 years) after initial diagnosis. Notably, the clusters showed major differences in survival rates (Fig. 4e). Large variability in survival between clusters occurred even when considering only one age group and gender (Supplementary Fig. 1). Distribution of laboratory measurements (Fig. 4f) varied between clusters. For example, the cluster 12 had only very small Troponine-T values, but most of the other clusters had wide range of Troponine-T values.

Our third example is depression, a growing problem among adolescents. In our material, analysis of the age- and gender-related incidence of depressive episodes (F32) demonstrated a sharp peak in female patients in their late teens, with the highest number at age 17 (Supplementary Fig. 2). Incidence was almost three-fold that of males at a similar age, and five-fold or more that of females over 30 or under 12. Reasons for this peak are apparently multifaceted, combining biological and societal factors, and also factors related to health care organization, which calls for further analysis.

## Discussion

In this study we show that data-driven diagnostic code-based clustering uncovers patient subgroups that show significant differences in diagnosis and demographic characteristics, in survival, and in potential biological associations. Our approach demonstrates that underutilized health-record information can help to identify patient groups needing different types of intervention, including follow-up or clinical care.

To exemplify, asthma is an umbrella diagnosis for several phenotypes such as allergic, non-allergic, and eosinophilic asthma. For such a polygenic multifactorial disease, grouping and categorization is generally challenging. One method for subcategorization is to identify disease-associated traits such as allergic sensitization, impaired lung function, or predisposition to exacerbations. By using diagnostic history data, we found 27 comorbidity clusters, in other words asthma subgroups. Although age was not a variable that we used for clustering, age-associations of diagnoses were often evident, as was association with mortality. We unexpectedly found large, statistically significant inter-cluster differences in some laboratory parameters, ones like eosinophils or renal function that require further assessment. These phenotypic differences may serve as a means to characterize new meaningful subgroups of asthma. Comorbidity clustering results in a more detailed picture of the patient’s clinical profile than does one diagnosis alone. For improved asthma subgrouping, we plan to extend the analysis by combining lung-function findings and data on exacerbations, medication, and environmental exposure, making hypothetically possible the detection of new asthma types. Moreover, many more comorbidities could be included than our current 100 diseases.

Atrial fibrillation has phenotypically different presentations. It usually starts with paroxysmal episodes, which become more frequent and long-lasting over time, finally evolving into chronic atrial fibrillation. The etiology of atrial fibrillation is still largely unknown, but myocardial fibrosis induced by various pathologic conditions plays an important role. Many common diseases linked to myocardial dysfunction, such as hypertension and heart failure, are comorbidities for atrial fibrillation^28^. Here, ICD10 comorbidity-based clustering of atrial fibrillation resulted in 31 groups. These clusters differed significantly in etiology and in mortality (Fig. 4a–e), but inside a quite narrow age range, in line with previous findings. Cluster 2 contained many young men without any distinct comorbidities. However, laboratory values such as C-reactive protein, the myocardial stress marker proBNP, and the myocardial injury marker troponin T showed significantly higher levels (FDR 0.1%) in Cluster 2 than in other clusters. This group would thus be very interesting for further study of background aspects of early-onset atrial fibrillation. Clusters need further evaluation and testing in prospective cohorts; early identification of patients in certain subgroups could guide clinicians in more personalized treatment and better outcomes.

Notably, the majority of patients (65%) had at least one concomitant diagnosis, and for 99 of the 100 diseases, our median number of diagnoses per patient was two or more. This demonstrates the impact of comorbidity in clinical practice. To individualize treatment based on the whole spectrum of disorders and while considering the distinct features, impacts, and interactions of each disease and medication is extremely challenging. Statistical models are therefore necessary, first in understanding and mapping populational heterogeneity and highlighting the significance of differing comorbidity profiles, and second in supporting decision-making.

For mapping a population’s phenotypic variability, large-scale, longitudinal, and multimodal data are essential. Large data repositories can help in detailing subtypes and rare associations not obvious in small cohorts or at individual patient level. Hospitals, biobanks, research institutions, and insurance- and governmental agencies worldwide already possess registries and data lakes. These are, however, utilized in medical research at a level far below their potential. The primary motivation for our work was to provide an overview of the possibilities that large-scale clinical data obtained in daily practice can provide for phenotyping. The usefulness of clustering has been shown earlier, but in specific diseases^16,27^, and by use of a few carefully selected variables that may not always be part of typical acquisition. Our study extends previous studies by the spectrum of diseases and diversity of data (longitudinal diagnostic data, survival data, and 100 of the most frequent laboratory tests selected uniquely for each index disease). For comprehensive analysis, we provide an online tool for browsing the extensive set of results.

Clinical data collections like those of this project are characteristically high-dimensional, longitudinal, incomplete, sampled at irregular intervals, and representing differing modalities and statistical distributions that challenge any methodology. Here, the chosen VAE model supported a discovery type of study, interpretability by visualization of populational structure in the two-dimensional latent space, and processing of a large amount of data with a reasonable computation effort. A weakness of our study is the origin of our health records; the records cover secondary-tertiary healthcare information but lack primary-care data.

In conclusion, longitudinal clinical profiles combined with advanced data analytics identified refined phenotypes in all 100 common disorders. We found that patients with the same underlying disease but differing comorbidity profiles have distinct mortality risks and clinical parameters, which could call for different therapeutic choices. Modeling the heterogeneity and the implications of differing patient profiles can advance individual health-risk assessment, treatment targeting, and follow-up strategies, as well as improve prognostication, best practices, planning of healthcare resources, and lead to etiological discoveries. Whereas most of the current treatment guidelines are based on clinical trials with stringent exclusion criteria for comorbidities, we demonstrate here with real-world data that comorbidity data linked to laboratory- and survival information can add to subgroup analysis a significant new level of information.

## Methods

### Diagnostic and demographic data

We retrieved ICD-10 codes, numerically expressed laboratory results, age, sex, date of last contact, and date of death from electronic health records of Helsinki University Hospital (HUS), using the data lake infrastructure that contains real-world data generated in the hospital, updated virtually in real time. HUS is a secondary-tertiary healthcare provider in all medical specialties that serves 1.7 million inhabitants in the Uusimaa region in Finland. The data were based on 1.28 million patients diagnosed during a 10-year period between 2009 and 2018. The 100 most common diagnoses (index diseases) encompassed 989,552 (77.6%) patients. We retrieved all diagnoses within a four-year follow-up period beginning from the first occurrence of the index disease in the database. To enable coverage of the full four-year follow-up period, we selected patients who had been diagnosed initially in 2015 or earlier. Further, to ensure reliability of the first date of index disease diagnosis, we selected patients who were not diagnosed with that specific index disease during a two-year period of 2009 and 2010. The total number of individual patients across the 100 datasets was 526,779, which we divided into 100 non-exclusive index-disease groups, each comprising from 4319 to 44039 patients (Fig. 1).

ICD-10 codes were expressed at the categorical level of three characters. As an exception, for asthma J45, codes J45.0, J45.1 and J45.8 were also extracted. Codes related to pregnancy and childbirth (O00–O99, P00–P96), malformations, and abnormal findings (Q00–Q99, R00–R99), external causes (S00–T98, V01–Y98), and health status and administration (Z00–Z99) we excluded^1^. Data quality we controlled by verifying patient uniqueness and correct ICD-10 formatting; entries not fulfilling the requirements we removed, with codes for symptoms and causes treated equally. Following the General Data Protection Regulation (GDPR), the cohort did not include patients who had denied the registry holder (HUS) the use of their data for research purposes. Identity information was pseudonymized, and dates expressed according to a relative timescale of days from birth.

Patients’ diagnoses in the follow-up period we expressed as a binary feature vector, in which vector elements indicated ICD-10 codes during the follow-up period. Those diagnoses with a prevalence less than 1% in the index group we discarded, resulting in final feature vector dimensionalities between 21 and 88, depending on index disease.

### Clustering

For robustness and reliability, cluster analysis with disease-specific binary feature vectors was done in two phases. The first phase included dimensionality reduction using a variational autoencoder model (VAE)^29^ followed by clustering in the continuous latent space of the model. VAE training and clustering took place first for a dataset that contained *N* patients with a specific index disease, and then we repeated the procedure independently 100 times after randomly subsampling *N*/2 patients at each run. The VAE model we implemented according to Keras documentation (https://keras.io/), and trained in 30,000 epochs. For simplicity, the dimensionality of the intermediate layer was at 40 and the latent representation at 2. Vectors in the latent space of the trained VAE model we clustered using a density-based HDBSCAN algorithm^30^ with a minimum cluster size (*min_cluster_size*) of N/100, and the parameter *min_samples* set at 5. No index-disease-specific optimization of parameters was done. The HDBSCAN algorithm includes outlier detection, and thus for some of the feature vectors not located in the dense regions, no cluster labels were assigned.

In the second phase, we used a modified version of a consensus index^31^ to quantify the robustness of the clustering that was done with all *N* patients*.* In short, corresponding to subsampled dataset *h*, let $M^{\left( h \right)}$ and $I^{\left( h \right)}$ denote *N* × *N* matrices, where the entries are defined as:

1 $$M^{\left( h \right)} \left( {i,j} \right) = \left\{ {\begin{array}{*{20}l} {1} \hfill & {\text{if items}}\,i\, {\rm and}\, j \,{\text {belong to the same cluster in dataset}}\,h, \hfill \\ {0} \hfill & {{\text{otherwise}}} \hfill \\ \end{array} } \right.$$

2 $$I^{\left( h \right)} \left( {i,j} \right) = \left\{ {\begin{array}{*{20}l} 1 \hfill & {\text{if items}}\,i\,{\rm and}\,j\, {\text{are present in the dataset}}\, h, \hfill \\ 0 \hfill & {{\text{otherwise}}} \hfill \\ \end{array} } \right.$$

Our consensus matrix represents the proportion of runs in which any two feature vectors (or patients) were assigned to the same cluster:

3 $$M\left( {i,j} \right) = \frac{{\mathop \sum \nolimits_{h} M^{\left( h \right)} \left( {i,j} \right)}}{{\mathop \sum \nolimits_{h} I^{\left( h \right)} \left( {i,j} \right)}}$$

The consensus matrix we constructed by using the cluster labels of the 100 subsampled datasets. Let $C_{k}$ denote indices of samples in the dataset of *N* patients assigned to cluster *k.* The consensus index with respect to cluster *k* has the form:

4 $$m\left( k \right) = \frac{1}{{N_{k} \left( {N_{k} - 1} \right)/2}}\mathop \sum \limits_{{\begin{array}{*{20}c} {i,j \in C_{k} } \\ {i < j} \\ \end{array} }} M\left( {i,j} \right)$$

The statistical significance (*p* < 0.001) of the consensus index we estimated using a permutation test. Null distribution was constructed by permuting cluster assignments of samples randomly 5000 times while keeping the consensus matrix fixed. Thereafter, feature vectors assigned to non-significant clusters we marked as outliers.

### Cluster characteristics

Diagnosis frequency within a cluster, as well as log_10_ odds ratio between a cluster and the rest of the patients (including outliers) we computed for each index disease cohort. Statistical assessment included 2 × 2 contingency table analysis (https://www.statsmodels.org/) with a 0.1% false discovery rate (FDR) using the Benjamini–Hochberg procedure^32^ in 174,144 comparisons across index diseases, clusters, and variables.

### Survival analysis

For each cluster of patients, survival function and 95% confidence were estimated by Kaplan–Meier analysis. Data utilized were the date of the first occurrence of an index disease, the date of the last encounter, and the date of death.

### Clinical laboratory data

Laboratory data collected from the database were limited to the 100 most common tests separately for each index disease. We selected for further analysis those measurements at the first occurrence of an index disease with a maximum of ± 6 months tolerance. Several numerical laboratory results we compared between patients assigned to a given cluster and the rest of the patients (per index disease), by using a two-sided Mann–Whitney U test with a 0.1% FDR (101,087 comparisons). The required minimum number of observations per laboratory test was set for both compared groups at 20. Moreover, for visualizing cluster-specific characteristics (online), we computed the common language effect size^33^, i.e. the probability that a randomly selected laboratory result is larger in patients in a specific cluster than in other patients.

### Ethical aspects

No ethical permission was required according to the Finnish Medical Research Act for the secondary use of medical records. Following national and EU legislation, the study was based on approval of HUS Helsinki University Hospital (permission HUS/466/2019).

## Supplementary Information

Supplementary Information.

## Data Availability

Due to national legislation, restrictions apply to the availability of clinical data at individual level, which were used with the permission of HUS Helsinki University Hospital. For data permission inquiries, please contact tietopalvelu@hus.fi.
