# Supplementary material for: Data-driven comorbidity analysis of 100 common disorders reveals patient subgroups with differing mortality risks and laboratory correlates
Source: Sci Rep. 2022 Nov 2;12:18492. doi: 10.1038/s41598-022-23090-3 (PMC9630271; doi:10.1038/s41598-022-23090-3)
Supplement: Supplementary file 1 — Supplementary Information. [file 41598_2022_23090_MOESM1_ESM.pdf]

Supplementary Table 1. 100 most common diagnoses.

Rank	Code	Description	Patients	Women				Men		
				%	Age, percentiles					
					25th	50th	75th	25th	50th	75th
**1**	I10	Essential (primary) hypertension	87,273	53	60	69	79	56	65	74
**2**	J06	Acute upper respiratory infections at multiple and unspecified sites	70,515	52	2	8	35	1	4	18
**3**	M54	Dorsalgia	66,696	59	34	48	62	35	48	61
**4**	J18	Pneumonia, organism unspecified	61,383	47	37	63	78	41	64	76
**5**	J45	Asthma	56,301	55	16	44	62	7	15	53
**6**	H25	Senile cataract	55,387	62	68	75	80	66	73	79
**7**	M79	Other soft tissue disorders, not elsewhere classified	54,662	60	34	50	63	30	49	65
**8**	F32	Depressive episode	54,168	63	16	19	37	17	25	45
**9**	I48	Atrial fibrillation and flutter	54,048	45	67	75	82	59	68	76
**10**	M17	Gonarthrosis [arthrosis of knee]	44,729	63	59	67	75	56	65	72
**11**	H66	Suppurative and unspecified otitis media	43,433	47	2	4	16	1	3	7
**12**	H90	Conductive and sensorineural hearing loss	43,345	54	34	59	73	19	58	72
**13**	G47	Sleep disorders	42,894	34	49	58	66	46	55	64
**14**	I25	Chronic ischemic heart disease	42,746	36	66	75	82	62	70	77
**15**	A09	Diarrhea and gastroenteritis of presumed infectious origin	40,501	54	6	32	65	3	23	55
**16**	F41	Other anxiety disorders	36,647	66	16	21	35	17	25	40
**17**	E11	Non-insulin-dependent diabetes mellitus	36,537	42	59	68	76	58	66	72
**18**	N39	Other disorders of urinary system	36,471	81	43	61	74	58	70	77
**19**	M25	Other joint disorders, not elsewhere classified	35,719	60	27	42	56	26	41	54
**20**	K57	Diverticular disease of intestine	33,404	59	58	67	76	53	64	73
**21**	M51	Other intervertebral disc disorders	33,299	54	38	48	59	38	48	57
**22**	I50	Heart failure	30,504	49	72	80	86	64	73	80
**23**	L20	Atopic dermatitis	30,063	54	5	21	39	2	8	30
**24**	N10	Acute tubulo-interstitial nephritis	30,058	64	19	54	76	48	66	77
**25**	K80	Cholelithiasis	29,707	65	42	57	70	49	63	74
**26**	I63	Cerebral infarction	29,281	47	60	72	81	55	66	75
**27**	M75	Shoulder lesions	28,915	53	47	54	61	47	55	62
**28**	K40	Inguinal hernia	28,275	14	35	63	76	46	62	72
**29**	F33	Recurrent depressive disorder	28,011	68	28	39	51	29	41	52
**30**	F10	Mental and behavioral disorders due to use of alcohol	26,849	33	25	42	55	35	47	57
**31**	E78	Disorders of lipoprotein metabolism and other lipidemias	26,712	46	56	66	75	52	62	71
**32**	I49	Other cardiac arrhythmias	26,429	57	45	66	78	46	65	75
**33**	H35	Other retinal disorders	26041	58	67	76	83	60	72	80
**34**	N30	Cystitis	25,769	84	28	59	78	55	69	79
**35**	L30	Other dermatitis	24,901	58	35	54	66	40	57	68
**36**	M16	Coxarthrosis [arthrosis of hip]	24,837	59	61	69	76	57	66	73
**37**	M23	Internal derangement of knee	24,772	50	35	48	58	32	44	53

**38**	C44	Other malignant neoplasms of skin	24,210	54	68	76	84	68	76	82
**39**	J35	Chronic diseases of tonsils and adenoids	24,004	54	7	18	30	4	8	23
**40**	E66	Obesity	23,852	60	33	45	54	18	47	57
**41**	N40	Hyperplasia of prostate	23,820	0				65	71	77
**42**	C50	Malignant neoplasm of breast	23,475	99	52	62	69	60	68	75
**43**	I21	Acute myocardial infarction	23,472	37	66	75	82	58	66	75
**44**	N92	Excessive, frequent, or irregular menstruation	23,435	100	37	44	48			
**45**	A49	Bacterial infection of unspecified site	23,392	49	51	66	77	52	66	75
**46**	I87	Other disorders of veins	23,133	70	44	55	67	48	58	69
**47**	J20	Acute bronchitis	22,790	52	25	52	70	5	45	69
**48**	H53	Visual disturbances	22,735	59	25	48	66	15	45	64
**49**	I70	Atherosclerosis	22,531	44	68	76	84	64	71	78
**50**	G56	Mononeuropathies of upper limb	22,342	65	44	54	64	45	55	64
**51**	G43	Migraine	22,164	74	21	34	47	12	18	41
**52**	H10	Conjunctivitis	22,084	55	10	32	53	5	18	46
**53**	J30	Vasomotor and allergic rhinitis	22,051	49	13	24	38	10	14	30
**54**	I84	Hemorrhoids	21,713	56	43	55	68	41	52	64
**55**	K59	Other functional intestinal disorders	21,174	57	6	25	63	5	9	48
**56**	K35	Acute appendicitis	20,937	49	25	38	57	24	36	52
**57**	H40	Glaucoma	20,809	61	61	71	79	55	68	76
**58**	D12	Benign neoplasm of colon, rectum, anus, or anal canal	20,761	50	58	67	75	59	68	75
**59**	M47	Spondylosis	20,670	57	54	63	72	51	59	68
**60**	J15	Bacterial pneumonia, not elsewhere classified	20,430	46	46	63	76	48	64	75
**61**	F43	Reaction to severe stress, and adjustment disorders	20,417	62	16	22	39	13	18	39
**62**	A46	Erysipelas	20,414	43	53	66	78	47	59	69
**63**	I20	Angina pectoris	20,211	39	64	73	80	60	68	76
**64**	J03	Acute tonsillitis	19,895	54	9	19	29	5	17	27
**65**	K21	Gastro-esophageal reflux disease	19,791	52	25	52	66	10	48	63
**66**	G45	Transient cerebral ischemic attacks and related syndromes	19,441	54	62	71	80	58	68	77
**67**	H65	Nonsuppurative otitis media	19,164	44	3	6	39	2	5	12
**68**	G40	Epilepsy	19,037	50	12	29	51	10	27	52
**69**	N83	Noninflammatory disorders of ovary, fallopian tube and broad ligament	18,934	100	33	45	59	21	26	26
**70**	J44	Other chronic obstructive pulmonary disease	18,900	40	62	69	75	63	69	76
**71**	M19	Other arthrosis	18,900	58	53	60	70	48	57	65
**72**	M48	Other spondylopathies	18,813	57	61	70	77	57	66	74
**73**	D25	Leiomyoma of uterus	18,548	100	41	47	52	30	43	43
**74**	G44	Other headache syndromes	18,317	70	29	42	56	31	43	55
**75**	C61	Malignant neoplasm of prostate	18,133	0				65	71	77
**76**	J31	Chronic rhinitis, nasopharyngditis and pharyngitis	18,089	53	30	45	58	26	42	56
**77**	D48	Neoplasm of uncertain or unknown behavior of other and unspecified sites	17,686	56	40	57	71	39	58	71

**78**	H43	Disorders of vitreous body	17,512	63	56	64	70	56	64	70
**79**	J21	Acute bronchiolitis	17,457	39	0	1	2	0	1	2
**80**	J01	Acute sinusitis	17,097	64	30	42	58	23	41	58
**81**	L57	Skin changes due to chronic exposure to nonionizing radiation	16,400	57	70	77	84	71	77	83
**82**	M20	Acquired deformities of fingers and toes	16,345	80	52	60	68	52	60	68
**83**	H26	Other cataract	16,206	67	62	75	82	47	64	77
**84**	K92	Other digestive system diseases	15,873	42	53	71	83	50	64	76
**85**	H04	Disorders of lacrimal system	15,462	70	41	57	70	32	51	67
**86**	J32	Chronic sinusitis	15,091	63	36	48	60	35	48	61
**87**	I35	Nonrheumatic aortic valve disorders	15,026	45	69	78	83	60	71	78
**88**	K02	Dental caries	14,736	43	16	45	64	18	48	64
**89**	N81	Female genital prolapse	14,566	100	57	68	76			
**90**	L50	Urticaria	14,361	62	22	39	53	8	27	49
**91**	K29	Gastritis and duodenitis	14,307	52	51	64	75	52	64	73
**92**	H02	Other disorders of eyelid	14,099	61	58	67	77	59	68	76
**93**	N97	Female infertility	13,884	100	30	33	36	31	31	33
**94**	H33	Retinal detachments and breaks	13,666	47	52	60	68	52	61	68
**95**	L02	Cutaneous abscess, furuncle and carbuncle	13,612	44	26	41	59	27	42	57
**96**	I80	Phlebitis and thrombophlebitis	13,604	57	42	59	72	45	57	69
**97**	A41	Other septicemia	13,570	48	38	61	74	40	60	71
**98**	D64	Other anemias	13,544	52	45	67	79	54	68	77
**99**	N84	Polyp of female genital tract	13,487	100	43	54	65			
**100**	E10	Insulin-dependent diabetes mellitus	13,443	44	14	28	48	14	30	51

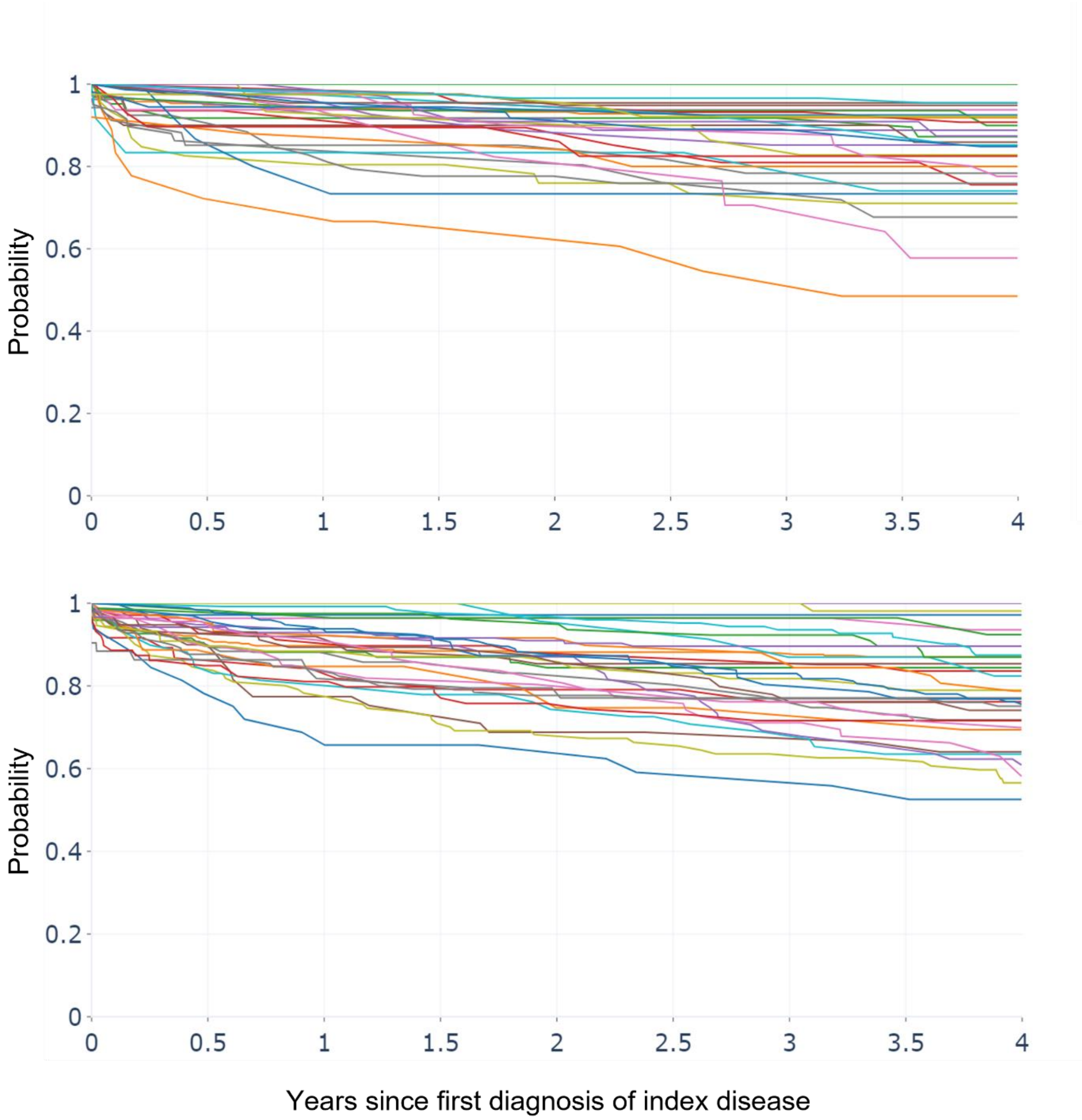

Supplementary Fig. 1. Atrial fibrillation. Cluster-specific Kaplan-Meier survival for 60- to 69-year-old women (upper) and men (lower).

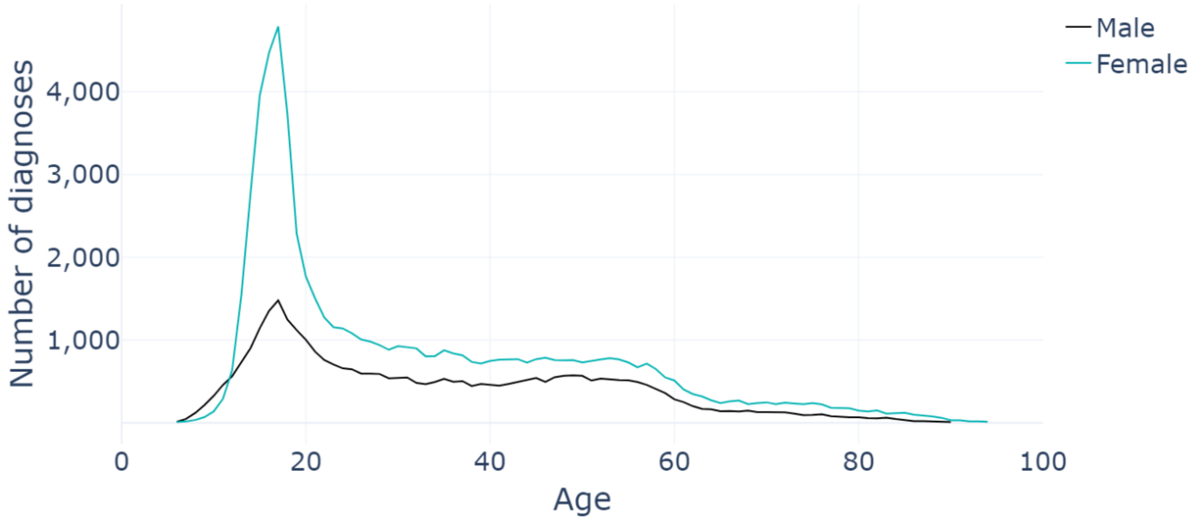

Supplementary Fig. 2. Number of depressive episode (F32) diagnoses by age.
